# Novel genetic findings

**DOI:** 10.1186/1746-160X-8-S1-I10

**Published:** 2012-05-25

**Authors:** Axel Bohring

**Affiliations:** 1 Institut für Humangenetik, Westfälische Wilhelms-Universität Münster, Germany

## 

Here we report on the clinical findings and key symptoms in *WNT10A*-related ectodermal dysplasias. The ectodermal dysplasias (ED) are a large, heterogeneous group of disorders characterized by defects in the morphogenesis of skin, sweat, sebaceous, submucous, and mammary glands, hair, nails and teeth. Numerous more or less distinct entities have been reported, however, most of them are very rare and the cause is often unknown. *EDA* mutations, resulting in hypohidrotic ectodermal dysplasia (Christ Siemens Touraine syndrome) are known as the most common cause of ED. Over the last two years, *WNT10A* has turned out to be the second major gene associated with ED. *WNT10A* mutation carriers present with a broad and variable spectrum of symptoms related to at least four known Mendelian conditions as there are autosomal dominant Selective Tooth Agenesis Type 4 and autosomal recessive Adontia of Permanent Dentition (APD), Odonto-Onycho-Dermal Dysplasia (OODD), and Schöpf-Schulz-Passarge syndrome (SSPS). In heterozygotes, penetrance is reduced and only about half of the carriers show a phenotype including mainly tooth and nail anomalies. Approximately 8% of the unaffected individuals in our control group carried a *WNT10A* mutation. Further studies are necessary. We conclude that *WNT10A* mutation analysis might become an important diagnostic test in many patients with selective tooth agenesis or severe oligodontia concerning the permanent teeth.

